# Snare traction-assisted ultra-long tunnel endoscopic resection of a giant exophytic cardia subepithelial tumor with diaphragmatic adherence

**DOI:** 10.1055/a-2706-3493

**Published:** 2025-10-02

**Authors:** Huihui Zhou, Huanqing Fan, Ye Zheng, Fengqin Zhu, Qian Zhao, Yaowen Zhang

**Affiliations:** 1 562122 Department of Gastroenterology, Affiliated Hospital of Jining Medical University, Jining, China; 2 562122 Department of Rehabilitation Medicine, Affiliated Hospital of Jining Medical University, Jining, China; 3 562122 Endoscopy Department, Affiliated Hospital of Jining Medical University, Jining, China

The incidental finding on the contrast-enhanced computed tomography scan ( Fig. 1 **a** ) of a 31-year-old man was as follows: an irregular soft-tissue mass located between the gastric fundus-cardia and the hepatogastric space. Gastroscopy ( Fig. 1 **b** ) revealed a submucosal bulge on the posterior wall of the cardia. Endoscopic ultrasound ( Fig. 1 **c** ) demonstrated a hypoechoic lesion arising from the muscularis propria, with clear margins and abundant peripheral blood flow ( Video 1 ). A therapeutic gastroscope fitted with a transparent cap was used to create a submucosal tunnel in the esophageal wall 3 cm proximal to the lesion on the posterior cardia wall, exposing a milky white tumor ( Fig. 2 **a** ). Dissection with an ITknife2 revealed an exophytic, complex, multifocal mass extending into the peritoneal cavity; standard tunnel vision was limited. An endoscopically introduced snare was tightened around part of the tumor with hemostatic forceps, and continuous traction toward the esophageal lumen ( Fig. 2 **b** ) provided panoramic exposure of the peritumoral plane. Meticulous dissection freed the tumor from surrounding connective tissue and severed its continuity with the gastric muscularis propria, while simultaneous right upper-quadrant needle decompression prevented pneumoperitoneum. Adhesions to the diaphragm were carefully released under traction. The intact specimen (≈ 8 × 2 cm, irregular) was extracted through the tunnel orifice ( Fig. 2 **c** ). Postoperative wound hemostasias was achieved ( Fig. 2 **d** ), and the tunnel entry was closed with seven metal clips. Histopathology confirmed leiomyoma. Postoperative recovery was uneventful, with no hemorrhage or perforation.

**Fig. 1:**
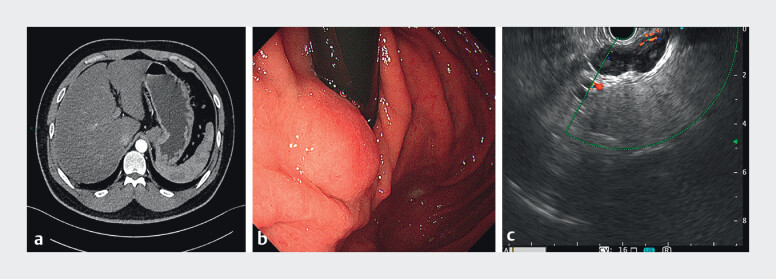
**a** The contrast-enhanced computed tomography shows an irregular soft-tissue mass located between the gastric fundus-cardia and the hepatogastric space. **b** Gastroscopy shows a submucosal bulge on the posterior wall of the cardia. **c** Endoscopic ultrasound shows a hypoechoic lesion arising from the muscularis propria.

Snare traction-assisted ultra-long tunnel endoscopy resection of a giant exophytic cardia subepithelial tumor with diaphragmatic adherence.Video 1

**Fig. 2:**
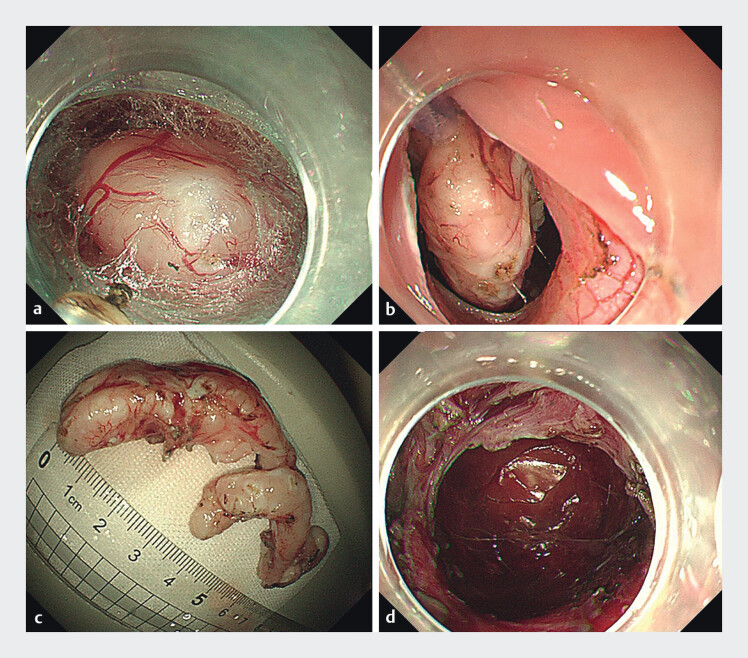
Endoscopic images. **a** A submucosal tumor with a milky white appearance. **b** Snare was tightened around part of the tumor. **c** The resected specimen, which was 8 × 2 cm in size. **d** Postoperative wound.

Classic submucosal tunneling endoscopy resection (STER) is limited to intraluminal lesions ≤3.5 cm in transverse diameter; tumor irregularity, deep origin, or operative duration >60 min are independent risk factors for complications 1 2 3 4 . Exophytic lesions >4 cm or with complex morphology have previously required combined laparoscopic-endoscopic approaches 5 . In this case, snare traction assistance provided complete exposure, enabling precise hemostasias and en bloc resection, shortened operative time, and reduced complications, thus offering a purely endoscopic solution for complex exophytic cardia lesions and extending the indications for STER.

Endoscopy_UCTN_Code_TTT_1AO_2AG_3AZ

## Notes

Endoscopy E-Videos https://eref.thieme.de/e-videos
*E-Videos* is an open access online section of the journal *Endoscopy* , reporting on interesting cases and new techniques in gastroenterological endoscopy. All papers include a high-quality video and are published with a Creative Commons CC-BY license. Endoscopy E-Videos qualify for HINARI discounts and waivers and eligibility is automatically checked during the submission process. We grant 100% waivers to articles whose corresponding authors are based in Group A countries and 50% waivers to those who are based in Group B countries as classified by Research4Life (see: https://www.research4life.org/access/eligibility/ ). This section has its own submission website at https://mc.manuscriptcentral.com/e-videos .

## References

[1] Chen T Zhou PH Chu Y Long-term outcomes of submucosal tunneling endoscopic resection for upper gastrointestinal submucosal tumors Ann Surg 2017 265 363 369 10.1097/SLA.0000000000001650 28059965

[2] Chen H Xu Z Huo J Submucosal tunneling endoscopic resection for simultaneous esophageal and cardia submucosal tumors originating from the muscularis propria layer (with video) Dig Endosc 2015 27 155 158 10.1111/den.12227 24444087

[3] Xiang AY Wang KH Su W Endoscopic resection of giant esophageal subepithelial lesions: experience from a large tertiary center Gastrointest Endosc 2024 99 358 3.7E13 10.1016/j.gie.2023.10.032 37852331

[4] Chai NL Li HK Linghu EQ Consensus on the digestive endoscopic tunnel technique World J Gastroenterol 2019 25 744 776 10.3748/wjg.v25.i7.744 30809078 PMC6385014

[5] Gonzalez JM Debourdeau A Philouze G Laparoscopic and endoscopic cooperative surgery for difficult resection of posterior esophagogastric junction gastrointestinal stromal tumors Endoscopy 2018 50 178 179 10.1055/s-0043-121136 29136672

